# Advances in the Structural Health Monitoring of Bridges Using Piezoelectric Transducers

**DOI:** 10.3390/s18124312

**Published:** 2018-12-07

**Authors:** Yunzhu Chen, Xingwei Xue

**Affiliations:** School of Traffic Engineering, Shenyang Jianzhu University, Shenyang 110168, Liaoning, China; cyz4187@163.com

**Keywords:** structural health monitoring, bridge monitoring, lead zirconate titanate (PZT), smart aggregate, reinforced concrete, grouting compactness

## Abstract

With the rapid development of the world’s transportation infrastructure, many long-span bridges were constructed in recent years, especially in China. However, these bridges are easily subjected to various damages due to dynamic loads (such as wind-, earthquake-, and vehicle-induced vibration) or environmental factors (such as corrosion). Therefore, structural health monitoring (SHM) is vital to guarantee the safety of bridges in their service lives. With its wide frequency response range, fast response, simple preparation process, ease of processing, low cost, and other advantages, the piezoelectric transducer is commonly employed for the SHM of bridges. This paper summarizes the application of piezoelectric materials for the SHM of bridges, including the monitoring of the concrete strength, bolt looseness, steel corrosion, and grouting density. For each problem, the application of piezoelectric materials in different research methods is described. The related data processing methods for four types of bridge detection are briefly summarized, and the principles of each method in practical application are listed. Finally, issues to be studied when using piezoelectric materials for monitoring are discussed, and future application prospects and development directions are presented.

## 1. Introduction

In recent years, with the rapid development of China’s transportation construction industry, many long-span bridges were built. With recent scientific and technological improvements at the industrial level, bridge construction technology experienced unprecedented progress [1]. Many bridges with a span of over 400 m were built in recent years, including 114 cable-stayed bridges and 109 suspension bridges. Among them, 59 cable-stayed bridges and 34 suspension bridges were built in China. More long-span cable-stayed and suspension bridges are under construction or planned for construction.

However, bridges may suffer from many defects, such as fatigue and brittle fracture, corrosion, instability, and mechanical damage. Therefore, bridge construction is extremely complicated and must be subject to strict monitoring. Technical personnel must perform calculations and measurements repeatedly. The stresses and displacements generated at the key sections and major control points of a structure are controlled within the scope of the design to ensure the safety and quality of construction. Monitoring various types of bridge structures is consistently an important topic in the field of engineering technology. Bridge health monitoring is related to the prosperity and stability of society and the safety of people’s lives and property [1]. Therefore, bridge monitoring is very important.

Environmental effects (wind, temperature, rain, earthquakes, etc.) and traffic loads can continue to affect long-span bridge behavior in the long term and lead to deterioration. Therefore, the study and application of bridge health monitoring and safety evaluation are very necessary for the sustainable development of bridge engineering. In the early 1990s, a number of important long-span bridges were equipped with a structural health monitoring system, not only to monitor the response of the structure, but also to monitor the environmental effects [2].

In a bridge monitoring system, strain gauges, fiber-optic sensors, acceleration sensors, piezoelectric transducers, and other sensors can be used to monitor the structure of various parts of a bridge. In recent years, due to their unique integrated functions of sensing and actuating, wide frequency range, fast response, simple preparation process, ease of processing, and low cost, piezoelectric material-based sensors were widely studied and applied in engineering. Piezoelectric transducers hold great potential in the health monitoring of structures [3,4,5]. Many scholars also used piezoelectric materials to carry out a large number of experiments on bridge monitoring and obtained certain results that attracted widespread attention. This paper reviews the progress of bridge monitoring based on piezoelectric materials [6].

## 2. Piezoelectric Material Introduction

A piezoelectric material is a crystalline material that appears in the voltage between two end surfaces when subjected to pressure. The direct piezoelectric effect and the inverse piezoelectric effect of the piezoelectric material can realize the mutual conversion of mechanical vibration (sound wave) and alternating current; thus, piezoelectric materials are widely used in sensor elements. Piezoelectric elements are subjected to force deformation such as thickness compression, length compression, volume compression, thickness shear, and plane shear [7]. Thus, a compressed piezoelectric material receives a signal by axial compression, and a sheared piezoelectric material receives a signal by shearing. Compressed piezoelectric material is mainly based on the d_33_ mode, and sheared piezoelectric material is mainly based on the d_15_ mode. Therefore, compression-type and shear-type piezoelectric sensors can be fabricated according to different working modes. Different modes of piezoelectric ceramic sensors also have different effects; for example, the piezoelectric ceramic of the d_31_ mode can be used to generate Lamb waves in a plate-like structure.

**Figure 1:**
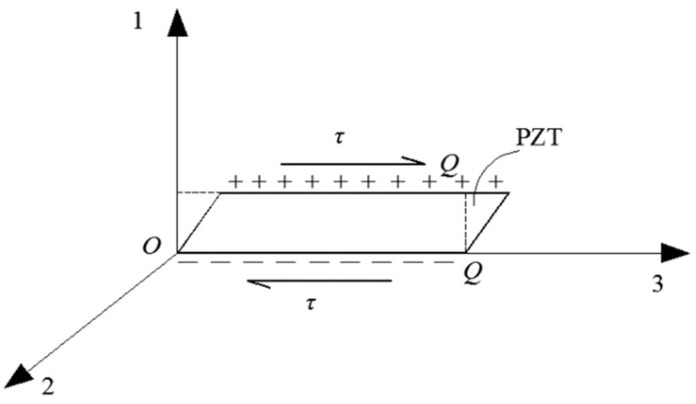
Direct piezoelectric effect of a shear-type piezoelectric ceramic [7,8].

Piezoelectric transducers are made using the direct piezoelectric effect. The direct piezoelectric effect describes how a piezoelectric material deforms when a stress is applied along a certain direction of the piezoelectric material. The two surfaces of the piezoelectric material generate charges of equal magnitude but opposite signs, which convert mechanical energy to electrical energy. Xu et al. [8] used d_15_-type shear piezoelectric ceramics to create a dynamic shear stress sensor using the direct piezoelectric effect. Figure 1 shows the direct piezoelectric effect of a shear piezoelectric ceramic, where *τ* is the shear stress of the shear piezoelectric ceramic, 3-3 is the polarization direction, and the plane perpendicular to the 1-1 axis is the electrode coverage.

## 3. Installation Types of Piezoelectric Transducers in Structural Health Monitoring

**Figure 2:**
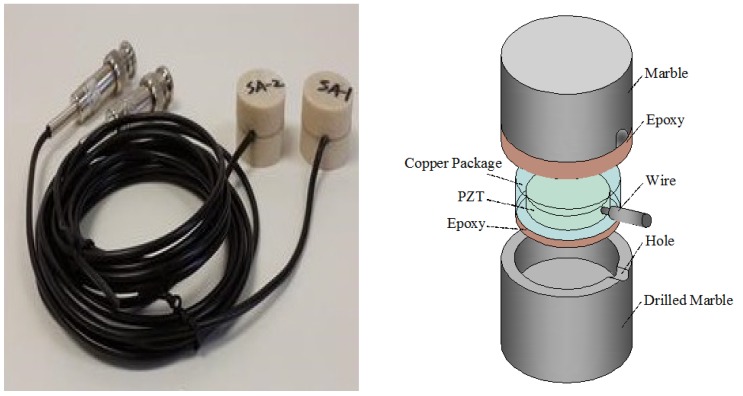
Illustration of smart aggregates [9].

Piezoelectric transducers can be externally bonded on the surface of a structure, or internally embedded in the structure to achieve structural health monitoring (SHM). The embedded piezoelectric transducers are fabricated by sandwiching a waterproofed lead zirconate titanate (PZT) patch with electric wires between two protective layers, which can be used as an actuator to generate a stress wave or a sensor to sense a stress wave. Smart aggregates (SAs) formed by piezoelectric patches and waterproof lead embedded in concrete blocks are a type of embedded piezoelectric transducer, as shown in Figure 2 [9]. PZT transducers can also be coupled to the surfaces of structures in ultrasonic experiments to receive stress waves and monitor the health status of those structures.

### 3.1. Bonded Paste

In recent years, polyvinylidene fluoride (PVDF) was widely used in SHM, and it can be bonded to the surface of the structure to monitor the health of the structure. In fact, PVDF is an externally bonded piezoelectric transducer [10]. Externally bonded piezoelectric transducers generally use piezoelectric ceramic transducers arranged in a matrix on the surface of the structure to monitor a range of impedance signals. Signals are processed using mathematical methods to obtain the damage index to determine the damage condition. The system features a simple sensor layout [10]. However, this type of arrangement may be affected by structural limitations and various factors. For piezoelectric transducers, the size, the position, and the instability of environmental conditions, such as temperature and humidity changes, as well as noise effects, affect its monitoring performance [11]. 

By comparing the impedance signals of different piezoelectric transducers and the root-mean-square deviation (RMSD) damage indicators, the externally mounted piezoelectric transducers can be used to identify multiple cracks, as well as for positioning and damage detection [12].

In the monitoring of structures using externally mounted piezoelectric materials, a bonding layer is required to connect the materials to the structure being monitored. Kaur et al. [13] studied the influence of the bonding layer and related parameters on the energy collection ability of PZT patches bonded to reinforced concrete structures with or without embedded structures. The authors presented a numerical model for the real dimensions of simply supported reinforced concrete beams with a surface bonded piezo-sensor (SBPS) and embedded PZT patches. Coupled-field analysis was performed on both configurations. A numerical model was used to study changes in load resistance and bonding, the piezoelectric thickness and the dimensions of the patch plane, and the effects of the presence of the shear modulus of the adhesive layer of the patch and a patch with generated voltage/closed power.

References [14,15] developed an electromechanical model that can be used to calculate the voltage and power generated by an SBPS and an embedded concrete vibration sensor (CVS). When CVS is installed in a reinforced concrete (RC) structure, the CVS operates in *d*_31_ mode; it is the composite material in manufacturing, has sound compatibility with the surrounding concrete, and can withstand the harsh conditions typically encountered in RC structures during casting.

The output of the PZT voltage is affected by four values, which are *S_1_* (longitudinal strain developed in the beam at the level of the PZT patch), *K_b_* (correction factor to take care of the shear lag effect in bond layer), *K_p_* (correction factor due to Poisson’s effect), and S_q_^*^ (circuit sensitivity), where the value of *K_b_* is independent of the material properties of the sensor and depends only on its geometry and the nature of the adhesive layer. *K_p_* is affected by the Poisson’s ratio of the main structural material and the piezoelectric coefficients *d*_31_ and *d*_32_. Under the other conditions, the larger the value of *d*_31_ is, the smaller the value of *K_p_* will be. The specific formula is described in detail in References [14,15]. Although the numerical models constructed for SBPS and embedded CVS are not the same, *Y_eff_*, which is width fraction for the PZT patch, is considered to be uniform for both SBPS and embedded CVS, ignoring the shear hysteresis effect along the PZT patch width.

Although the external bonding conditions may produce a higher voltage, from the perspective of the piezoelectric parameters, the embedded structure is more desirable, especially when the thickness is greater. In addition, experiments showed that the adhesive layer covering the surface of the patch for adhesion not only provides the protection for the patch, but is also very beneficial for outputting higher voltage and higher power.

### 3.2. Internal Embedded

PZT piezoceramic materials are lightweight, have little influence on the bulk structure, and can be embedded inside a newly constructed structure for SHM. Such materials provide a new method for the non-destructive evaluation of structures. The embedded piezoelectric transducers are better suited for detecting tiny damage inside the concrete structure than the surface-bonded type when the concrete structure is large. Embedded PZT piezoelectric ceramic structures can more accurately and comprehensively monitor the damage of structures; thus, the use of the embedded PZT transducers became an effective method for large-scale structural health monitoring [16]. In addition, this approach can overcome the shortcomings of PZT vulnerability and provide protection for the core of PZTs for better structural health monitoring and other purposes [9].

Song et al. [9] proposed a multifunctional smart aggregate that had three main roles: early strength concrete monitoring, impact detection, and structural health monitoring. Prior to casting a concrete structure, the proposed smart aggregate is embedded in the desired location. The development of concrete strength is monitored by observing the high-frequency harmonic response of smart aggregates. The effect on a concrete structure can also be detected by observing the open-circuit voltage of the piezoelectric ceramic patch in a smart aggregate. SAs were also used in the study of reinforced concrete beams [17,18,19], columns [20], shear walls [21], frame structures [22], fiber-reinforced polymer (FRP) repair concrete columns [23], and bridges [24]. Tsangouri et al. [25] used embedded piezoelectric sensors to seal cracks and monitor damage recovery in concrete repair systems.

Huo [26] proposed the dynamic transfer of the application of intelligent aggregates. In this context, it is necessary to establish a corresponding mechanical model to analyze the factors influencing the monitoring effect or simulate the monitoring effects to improve practical application. Therefore, Huo proposed the incorporation of a dynamic waterproof layer and the embedment of a protective layer in a PZT transducer model. The accuracy of the model was verified by the finite element method, and the influence of the protective layer, waterproof layer, and PZT patch on the dynamic performance of the sensor was analyzed. The system’s performance was also verified based on dynamic stress transfer experiments of PZT transducers.

**Figure 3:**
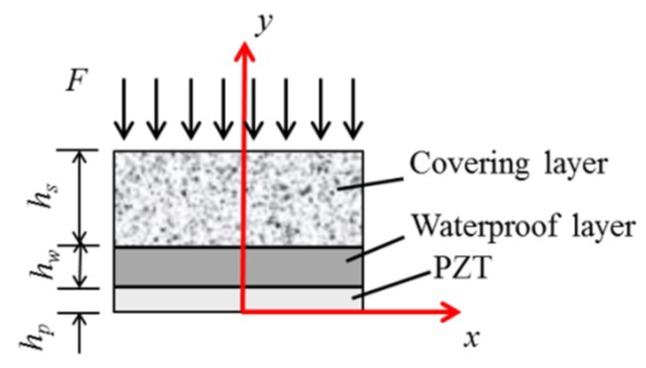
A half-model of the embeddable lead zirconate titanate (PZT) transducer [26].

A PZT patch with waterproof and protective layers can be used as an embedded PZT transducer for the damage detection of concrete structures, as shown in Figure 3.

The function of the waterproof layer is to provide electrical protection for the PZT patches, while the protection layer provides mechanical protection for the PZT patch. This type of embedded PZT transducer tends to use the *d*_33_ mode—the compression mode—to measure stress and to obtain stress waves in the polarization direction of *d*_33_. The vibration mode of the PZT transducer is expressed as

(1) $$\frac{dU_{p}}{dy}=C_{1}{Г}_{P}cos\left({Г}_{P}y\right).$$

The strain of epsilon P along the *Y*-direction is derived as

(2) $$\varepsilon_{p}=\frac{2}{h_{p}}C_{1}sin\left({Г}_{p}h_{p}\right)e^{i\omega t}.$$

There is a linear relationship between the output voltage and the strain along the polarization direction of the PZT patch. The output voltage of PZT can be expressed as

(3) $$V=\frac{\varepsilon_{p}d_{33}A}{s_{33}C},$$

where *d*_33_ is the piezoelectric constant of the PZT transducer, *S*_33_ is the elastic coefficient of the PZT transducer, and *C* is the capacitance of the PZT transducer.

### 3.3. Piezoelectric-Based Intelligent Interface

In addition to the above two installation types, there is also an installation technique to overcome the major issues related to pre-determining effective frequency bands for the piezoelectric-based SHM, namely a piezoelectric-based smart interface. In the study of many impedance-based SHMs, an important issue was raised, i.e., how to identify the effective frequency band of the impedance characteristics of the preload changes. In practice, the effective frequency band is usually determined by trial and error because it depends on the local dynamics of the structure being monitored [27]. Installable interface technology can be a potential solution to the above problems [28].

**Figure 4:**
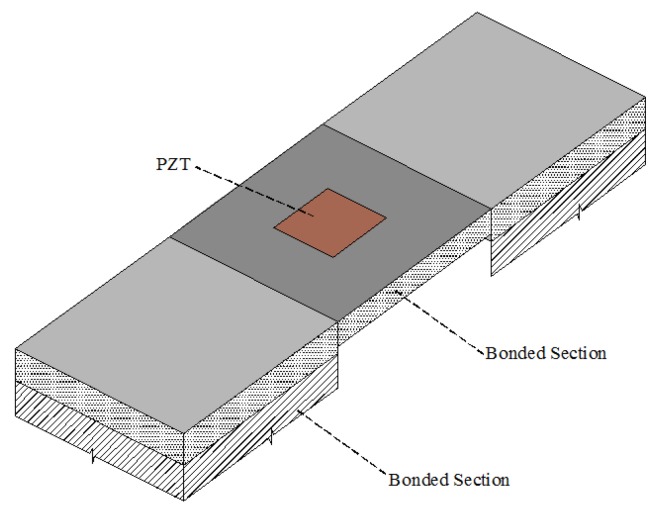
Prototype of PZT interface [27].

This technique indirectly acquires the sensitive impedance data from the target structure using an interfacial structure equipped with a piezoelectric sensor. As shown in Figure 4, the PZT interface prototype has a plate-like structure with two outside bonded sections and a flexible intermediate section, in which a PZT sensor is embedded. The flexible section can provide the free vibration subjected to the excitation of PZT. Due to the presence of the bonded sections, the PZT interface prototype can be installed and easily reconfigured when needed. Based on this installation technique, Huynh et al. [27] developed an impedance monitoring method based on the PZT interface for monitoring bolted connections. Nguyen et al. [29] used the electromechanical impedance-based method to monitor tendon anchorage of a cable-stayed bridge with a smart PZT interface. Huynh et al. [30] also studied how to monitor the tendon-anchorage impedance through an installable PZT interface with temperature compensation.

## 4. Data Processing Methods for SHM with PZT

When monitoring a bridge, some data processing methods are necessary to extract the variety of available information from the resulting signal. Common data processing methods include the wave method, time inversion, acoustic radiation, and impedance analysis.

### 4.1. Wave Analysis

Stress waves are usually used for structural damage detection and health monitoring [24,25,26,31,32,33,34], while PZT transducers are usually used to generate and detect stress waves. Li et al. [35] built a piezoelectric smart concrete active health monitoring system based on wave theory by embedding piezoelectric actuators and sensors in a concrete standard test block and transmitting and receiving stress waves in concrete. The uniaxial failure test of a smart concrete standard specimen yields changes in the shear wave velocity, relative energy attenuation, and frequency domain characteristics with damage.

A crack damage monitoring method based on the piezoelectric wave method was proposed for concrete structures by Sun et al. [36]. The mechanism of the stress wave of concrete cracks was analyzed. Based on this premise, the method of discriminating between different degrees of damage was proposed. At the same time, based on the characteristics of a given concrete structure, the sensor array can be used in the method of approximate damage location.

The researchers investigated the relationship between the damage location and the RMSD of the same structural admittance of a piezoelectric ceramic (PZT) under the same degree of damage, location of the damage, and PZT. They firstly built a concrete damaged beam model with a PZT sensor, then numerically analyzed the changes in the concrete beam before and after various forms of damage, and evaluated the degree of damage using RMSD indicators. The results showed that, under the same degree of damage, the RMSD index of damage determination decreases with the increase in the distance between the damage location and the PZT; when the distance between the damage location and the PZT is the same, the RMSD index increases with the degree of damage. Thus, there is an increasing trend [37]. 

Some scholars used wavelet neural networks to monitor bridge health. Based on their advantages in addressing complex nonlinear problems, wavelet analyses and neural networks were applied to the two technical problems of linear monitoring and stress monitoring of large bridge structures. The results showed that the wavelet neural network model has high monitoring accuracy and provides technical support for the safe operation of bridges [38,39]. Chen combined the piezoelectric wave method with the early strength theory of concrete to study the relationship between the early strength of concrete and the energy of the wave signal, and obtained a fitting equation for the strength percentage and the energy ratio of the strength grade of concrete specimens. The method was used to monitor and predict the early strength of concrete based on the piezoelectric wave method [40].

The time reversal method is an adaptive focusing method with unique advantages, which is of great value for the elimination of image distortion in ultrasonic imaging [41]. Ultrasonic guided wave time reversal processing of structural health monitoring can make complex signals realize signal focusing or reconstruction, and the higher the number of participating sensors is, the more obvious the focusing effect will be; moreover, the higher the signal complexity is, the more contrast there will be between the signals before and after focusing [42]. In health monitoring based on piezoelectric ceramics, many scholars conducted in-depth research, for example, using the time reversal method to monitor the pre-tightening force of the rock bolt [43], monitoring the health of the cup-shaped stent joint connection [44], monitoring the filling density [45], etc.

To suppress the influence of noise on reconstructed images, a time reversal imaging (TRI) algorithm based on noise suppression was proposed by Gao [46]. This method uses the cross-correlation of the time reversal signal as an imaging function to detect the damage of a plate structure. Because the noise is independent of the TR focusing signal, the algorithm can effectively suppress the noise even when very few echo signals are available. Therefore, the proposed method can effectively suppress noise. The experimental performance of the proposed method was compared with that of three traditional methods. Imaging results showed that even under low signal-to-noise ratio (SNR) conditions, the proposed method could clearly reveal the damage of two samples with high spatial resolution. In contrast, the damage location calculated by the three traditional methods was submerged in noise and could not be distinguished.

Zhang et al. proposed a time reversal method to monitor the looseness of cup-lock connections using active sensing based on stress waves. The peak value of the time reversal (TR) focusing signal can be used to monitor the tightness of the cup-lock connection. By comparing the trend curves of the peaks of channels 1 and 2, it was found that the peaks of the two channels steadily increased as the tightness of the joint increased. In addition, the experimental results showed that the TR method is superior to the energy method in terms of consistency, sensitivity, and noise immunity [45]. The method of Tian and other methods were used to monitor the quality of grouting through time inversion, which can quantitatively indicate the existence of grouting [47].

### 4.2. Acoustic Emission (AE)

All types of bridges in the world are aging, requiring maintenance and reinforcement. Therefore, it is urgent to provide long-term continuous monitoring techniques and methods for actual structures. As an important non-destructive testing technology, AE technology, supported by modern high-capacity computers, became a reality for the dynamic monitoring of bridges and other large buildings [48].

Extensive studies on the fracture and failure of concrete show that concrete should be regarded as a quasi-brittle material with size-dependent behavior. Many experimental techniques were used to evaluate fracture processes, and some modeling methods were developed to predict fracture behavior [49,50]. AE technology has the potential to effectively monitor the integrity of large structures, such as civil engineering structures, through a limited number of sensors [51]. The non-destructive method based on AE technology was proven to be highly effective, especially in evaluating and measuring the structural damage that occurs under mechanical loads. Carpinteri et al. compared the acoustic emission frequency statistical characteristics of a material with a defective size distribution during the damage process. AE technology was applied to the structural components monitored during the damage process. By quantifying the *b*-values at each loading stage, the correlation between the number of AE events and their amplitude was established. At the same time, the parameters beta and T were determined on a fixed time scale [52].

Huo et al. [53] reported a method of using a piezoelectric transducer as a strain gauge and an acoustic emission sensor simultaneously. Low-frequency signals are used to measure strain, while high-frequency signals are used as acoustic emission signals related to local damage. The acoustic emission signals in piezoceramic transducers are processed using *b*-value theory. The experimental results showed that the low-frequency signal extracted from the piezoceramic transducer was in good agreement with the frequency signal of the strain meter, and the high-frequency signal could be produced by the piezoceramic transducer, as the acoustic emission could indicate the local damage of the concrete. The experimental results verified the feasibility of using a piezoceramic sensor as a strain meter and acoustic emission sensor for structural health monitoring, which can promote applications in civil engineering.

In addition to the acoustic emission monitoring of the main structure of a bridge, many scholars explored the monitoring of the damage of key components, such as steel bars and bridge suspension cables [54]. Fricker and his colleagues [55] applied acoustic emission techniques to monitor the damage of pre-stressing tendons in a pre-stressed reinforced concrete bridge in Switzerland. The monitoring results were in accordance with the actual damage of the structure. Yuyama et al. [56] used acoustic emission technology to monitor the damage of post-tensioned pre-stressed beams. Through the analysis of acoustic emission signals, damage due to the corrosion and fracture of steel bars was identified and accurately located. Mohammed [57] carried out a fracture test of bridge hangers and cables in the laboratory, compared acoustic emission technology with other methods, and created a device to dynamically monitor changes in the steel wires of cables. Paulson [58] studied the application of acoustic emission technology to the long-term monitoring of the main cable and cables of a suspension bridge, successfully separating the acoustic emission signal from other signals and noise signals, and provided a reference for studying the long-term damage of bridge cables. Piervincenzo et al. [59,60] used acoustic emission techniques to monitor the damage of carbon fiber cables. The results showed that acoustic emission technology could be used to monitor induced damage and the development thereof more accurately than other methods. The test results obtained for parallel carbon fiber bundles and carbon fiber strands showed that the corresponding relationship between the acoustic emission activity and damage degree was good before damage completely destroyed the fibers.

### 4.3. Electromechanical Impedance (EMI) Analysis

In EMI technology, when a PZT transducer is subjected to high-frequency structural excitation in the presence of an electric field, the PZT transducer interacts with the bulk structure to create a unique health characteristic as an inverse function of the structural impedance. Using the automatic and inductive functions of the PZT transducer, the EMI model attempts to detect the load and damage of the structure to be monitored [61]. Compared to traditional non-destructive testing methods, EMI technology has several advantages. Firstly, due to the high-frequency range employed, the method is very sensitive to initial damage in the structure; secondly, it is unaffected by changes in boundary conditions, loads, or operational vibrations.

As mentioned above, the electromechanical impedance method is a type of health monitoring technology for real-time diagnosis and is particularly sensitive to local initial damage [62]. For example, for the fatigue problem, many scholars used EMI technology. Giurgiutiu et al. [63] showed that the EMI technology and the Lamb wave technology using piezoelectric active sensors can detect the existence and propagation of cracks under mixed-mode fatigue loading. Ihn and Chang [64] used wave propagation technology to detect damage using built-in PZT patches. They demonstrated that the technique can effectively detect stripping or delamination and monitor fatigue crack growth [65]. Soh and Lim [66] used EMI technology to study the feasibility of fatigue-induced damage detection and characterization. Experimental studies on aluminum beams with pre-induced circular notches showed that EMI technology is excellent for detecting fatigue cracks by varying the admittance characteristic frequency. Lim et al. [67] studied the feasibility of using EMI technology to detect micro-cracks invisible to the naked eye based on the work of Soh and Lim, and analyzed the sensitivity of different frequency ranges. A qualitative critical crack identification method was also proposed.

Park et al. [68] conducted an experimental study on how EMI technology can be used to detect structural damage in real time, and described a modified frequency-domain autoregressive model with exogenous inputs. Park et al. [69] proposed a new SHM technology that uses both high-frequency structure excitation and the response of a piezoelectric sensor to monitor local areas of the structure to reveal changes in structural impedance to indicate impending damage. Shin et al. [70] studied the role of EMI sensing technology in early concrete on-line strength gain monitoring using externally mounted piezoelectric sensors, and found that EMI characteristics are very sensitive to the intensity gain in early concrete.

An electromechanical impedance (EMI) model accounting for the shear lag between a PZT patch and a host structure was presented by Yang et al. [71]. Piezoelectric materials are commonly used in the form of sheets, and the damage state of a structure is judged by measuring the change in electrical impedance. In recent years, increasing research was conducted on the application of piezoelectric impedance technology for structural health diagnosis. Quinn and Lopes et al. [72,73] used a new method to analyze electromechanical impedance spectra and study the identification of structural damage.

### 4.4. Compensating Temperature Effects

The dynamic characteristics of the bridge and the piezoelectric properties of the transducers are temperature-dependent, and bridge structures exist in varying environmental conditions. Hence, temperature-compensated techniques are necessary for data processing and decision-making to obtain reliable and accurate SHM results.

It is well known that changes caused by environmental influences, such as temperature, often mask more subtle structural changes caused by damage. Sohn studied a linear adaptive model to distinguish the changes of modal parameters caused by the temperature variation, the structural damage, and other environmental influences, and the proposed model was verified using the monitoring data of the Alamosa Canyon Bridge [74]. Soh proposed a possible solution to create a constant temperature environment in the surrounding environment of the PZT patch, which is feasible for places with little variation of annual environmental conditions [75]. Huynh proposed a cross-correlation-based temperature-effect compensation algorithm using an effective frequency shift (EFS) of impedance signatures [30]. Baptista et al. experimentally studied the effect of temperature on the electrical impedance of the piezoelectric sensors used in the EMI technique, and the results showed that the temperature effects were strongly frequency-dependent [76]. Baptista [77] proposed a simple universal measurement system based on EMI technology for SHM, in which the data can be acquired in real time from multiple sensors, and the temperature effects can be compensated. Koo proposed a new damage detection strategy based on the correlation coefficient between the reference impedance data and the concurrent impedance data with effective EFS. In order to reduce the influence of temperature, an outlier analysis for the optimal decision boundary for damage detection was performed [78]. Based on this research, Koo also developed an automatic continuous monitoring framework using MATLAB and combined it with current hardware systems. Verification of the proposed technique was also carried out on laboratory-sized steel truss bridge members in a temperature-changing environment, and an outlier analysis was also performed that provided optimal decision constraints under unavoidable changes [79].

## 5. Application of Piezoelectric Materials in Bridge Monitoring

### 5.1. Monitoring the Concrete in Bridges

When monitoring bridges, concrete is the key component to be monitored in order to prevent the occurrence of defects such as cracks in concrete structures or slipping between steel and concrete, which endangers bridge safety.

The cracks produced in concrete are extremely harmful to the stability of bridges, and piezoelectric ceramic sensors can monitor the occurrence of concrete cracks. The direct and inverse piezoelectric effects of piezoelectric ceramics enable them to be both transducers and actuators. Zhao et al. [80] placed a piezoceramic into concrete and stimulated it to produce stress waves in concrete to realize the monitoring and identification of micro-crack damage in concrete.

Dumoulin et al. used an embedded piezoelectric sensor to perform ultrasonic monitoring of concrete cracks. The use of an embedded transducer improves the efficiency and repeatability of ultrasonic testing compared to conventional ultrasonic transducers, and enables measurements to be made at inaccessible locations during structural use [81].

**Figure 5:**
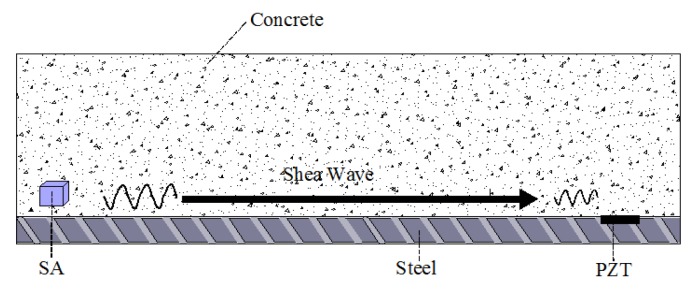
Demonstration of the shear wave-based active sensing approach used in a concrete-encased composite structure [82].

**Figure 6:**
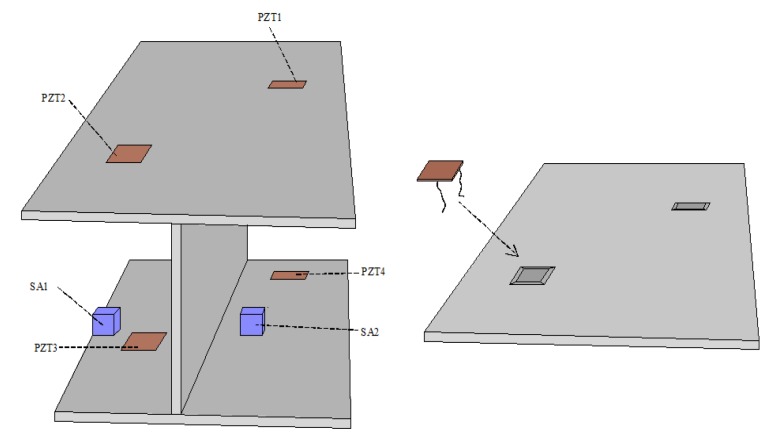
Installation of PZT sensors [82].

Reinforced concrete is an essential part of a bridge; however, concrete-encased composite structures show improved strength, ductility, and fire resistance compared with traditional reinforced concrete. Thus, such materials can also be applied in bridge construction. Despite their many advantages, however, one major disadvantage of this type of structure is the bond slip introduced between steel and concrete, which directly reduces the load capability of a structure. Therefore, Lei Zeng et al. [82] studied and developed an active sensing method using shear waves to monitor and predict the development of bond slip in concrete-encased composite structures (shown in Figure 5). As shown in Figure 6, the distributed piezoceramic sensor installed in the cavity of the steel plate acts as a sensor, and detects the wave response of a shear mode smart aggregate. This method can be used to determine the occurrence and development of bond slip in concrete-encased structures. In addition, M. Saafi et al. [83] used the active damage inquiry (ADI) method via a piezoelectric transducer array attached to structures to detect and locate the debonding and delamination of advanced composite reinforced materials from concrete structures.

### 5.2. Monitoring Bolt Looseness in Bridges

Bolts are widely used in bridge construction, and the connection state of bolts has an important influence on the safety and reliability of entire structures. However, failure due to bolt loosening or pre-loading may result in the failure of bolted connections and jeopardize the normal operation of a structure. Therefore, it is beneficial to monitor the health of bolted connections in real time. Indeed, many scholars studied this issue. At present, the electromechanical impedance method involving a piezoelectric ceramic transducer [84,85,86,87] was used to monitor the looseness of bolt connections [88,89]. In addition, the implementation of the active sensing method using a piezoelectric ceramic transducer gained increasing attention in SHM, because damage in a structure usually weakens wave propagation [17,90,91,92]. Yang et al. [93] used ultrasonic waves to monitor bolt loosening. When a wave propagates through the bolt connection surface, the energy dissipation is influenced by the integrity of the interface based on the contact mechanics. Wang et al. [94] studied the signal energy received by a piezoceramic patch when an ultrasonic wave passed through a bolt connection interface under different pre-loading conditions. The response wave energy was determined as proportional to the axial load of the bolt, allowing for the bolt to be monitored.

Huo et al. [95] developed an effective method of bolt connection health monitoring to detect bolt pre-tightening loss or bolt loosening, which leads to bolt joint failure. Based on fractal contact theory, a new model of bolted connection analysis was presented, and a pair of piezoelectric ceramic transducers was used to generate and detect ultrasonic waves. The research of Huo et al. was based on the inherent contact mechanism between two contact surfaces to achieve more accurate quantitative monitoring of bolt loosening.

**Figure 7:**
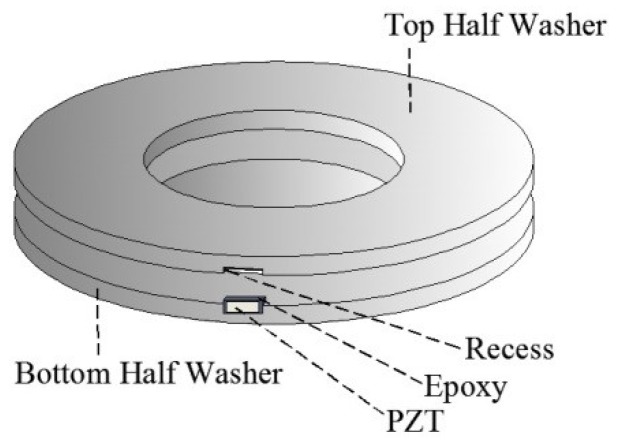
The design of a smart washer (SW) [97].

Because tangential damping has an important influence on wave propagation through the energy dissipation of bolted connections, different degrees of pre-loading are needed. Based on fractal contact theory, Wang et al. [96], considering the energy dissipation of the tangential damping of a bolt connection under the pre-tightening force of different bolts, took into account the effect of an incomplete interface and directly realized accurate quantitative monitoring. For bolt looseness monitoring, Huo et al. [97] also proposed a “smart washer” (as shown in Figure 7), manufactured by embedding a piezoelectric ceramic patch into two prefabricated flat metal rings and using it as a sensor to detect the loosening of bolt connections. It is possible to establish the relationship between the degree of any pre-loading degradation of bolt connections and the response signal of the stress wave propagating between the two washers. In addition, the pre-load loosening index based on the wavelet energy ratio is used to reflect the pre-load loss of bolts.

Huo et al. [98] also developed an electromechanical impedance (EMI) method based on smart washers (SWs) to study the pre-load conditions of bolted connections. Kong et al. [99] proposed a new non-destructive testing method based on impact to determine the health status of bolted connections by means of machine learning. This method is very similar to the percussion diagnostic technology used in clinical diagnosis for patients’ health and automatically detects and determines the health of a structure.

### 5.3. Monitoring of Corrosion of Steel Bars in Bridges

Because of the widespread application of reinforced concrete in bridges, the performance of reinforced concrete directly affects the performance and duration of a bridge. Therefore, steel bar corrosion is a key aspect in bridge monitoring.

A new type of encapsulated cement-based piezoelectric sensor was developed. The corrosion conditions of a steel bar can be determined from the relative amplitude of the signal measured by a piezoelectric sensor [100]. Li [101] used the direct and inverse piezoelectric effects of piezoelectric ceramics to embed an electromechanical impedance sensor at a specific location in concrete and applied an alternating electric field to the sensor. By analyzing the relationship between impedance and frequency, the corrosion of steel bars in a coagulant structure can be identified and monitored in comparison with the electrical impedance spectrum obtained under no-damage conditions.

Compared with traditional sensor systems, mechanical and electrical impedance sensors, such as those built by Li et al., are more adaptable to concrete structures, demonstrating good compatibility between the sensors and concrete structures. The durability of these sensors is better than that of other sensors because of the waterproof material used to protect them; furthermore, the sensors are buried within structures to prevent external interference.

### 5.4. Monitoring the Damage of Steel Pipes and Concrete in Bridges

**Figure 8:**
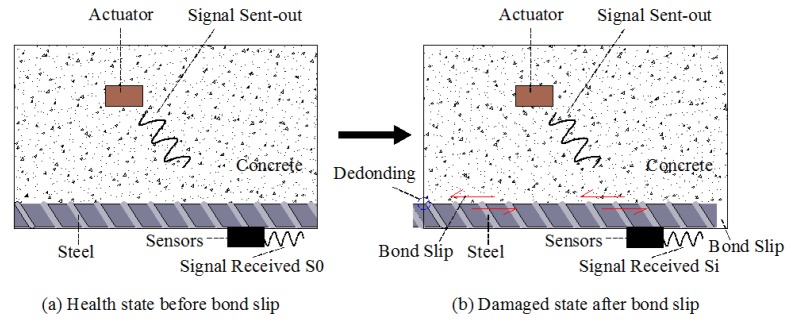
Smart aggregate-based active sensing approach to detect bond slip between the steel plate and concrete [104].

FRP is a composite material with high corrosion resistance and a high strength-to-weight ratio. This material was used increasingly in reinforced concrete structures [102]; thus, it was also used in bridge structures. The effectiveness of a structure depends on the bond between FRP composites and concrete structures. Therefore, the detection of debonding between FRP materials and bearing concrete structures is very important for ensuring the safety of bridge structures. Jiang et al. [103] proposed an active sensing method based on stress waves to monitor the debonding process of FRP tendons carrying concrete structures. Qin et al. [104] developed an active sensing method, as shown in Figure 8, using piezoceramic sensors to monitor the development of bond slip between steel and concrete.

In the construction of bridges, drainage is also very important. In the selection of drainage pipes, reinforced concrete drainage pipes are widely applied. However, cracks and leakage are the major causes of drainage pipe structural failures, which directly lead to economic losses and environmental damage. Feng et al. [105] proposed an active sensing method based on piezoceramic to detect cracks and further leakage of concrete pipes. The authors studied circumferential and axial cracks. When a stress wave propagates along a concrete pipe, the attenuation of the stress wave energy is sensitive to the direction of crack formation. Based on the wavelet packet energy analysis method, the type of crack can be identified, and further leakage due to cracks can be determined based on the energy attenuation of different stress waves propagated by cracks. Park et al. [62] carried out an impedance-based technique to detect damage in a sample tube with bolted connections. Lay-Ekuakille and others studied pipeline leakage detection using the impedance method [106]. Mary proposed a monitoring system based on a wireless sensor network (WSN), which is suitable for the remote monitoring of pipeline structures [107]. Wang and Chen [108] developed a distributed computer system/network for detecting and locating leaks caused by illegal drilling on pipelines. Guo and Du [109] designed a new type of sensor and proposed a proper Lamb wave mode to detect anomalies inside a long tube wall at a specific depth. There are many pipeline structures in the load-bearing members of the bridge, and the monitoring method of drainage pipelines can also be applied.

Additionally, concrete-filled steel tubular (CFST) members are widely utilized in large-span bridges. The effective restraint of steel pipes on core concrete is the key to exerting its mechanical properties. The peeling of the inner wall of steel pipes and the core concrete will have a negative impact on the bearing capacity of the members. Cai et al. [110] used a piezoelectric ceramic sensor bonded to the outer wall of the steel pipe, and, through the analysis of the frequency response function of the piezoelectric ceramics in the appropriate frequency band, the monitoring of the peeling damage of the CFST interface was realized. Xu et al. [111] also used an externally mounted piezoelectric ceramic sensor to analyze the output signal of the piezoelectric sensor through a wavelet packet to accurately identify the interface damage area of the CFST test model.

### 5.5. Monitoring the Strength of Concrete in Bridges

Concrete strength is also a concern regarding the quality of bridges. It is well known that the strength of concrete increases with age over a certain period. Many researchers also proposed different methods for monitoring concrete strength.

**Figure 9:**
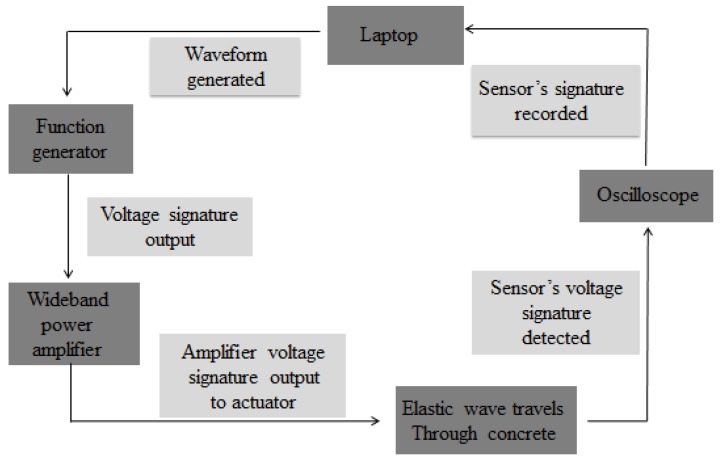
Typical experimental set-up of the wave propagation (WP) technique [112].

It is essential to evaluate the quality of concrete in construction and evaluate strength development during maintenance. In particular, early age maintenance intensity monitoring is very important for reducing construction costs and construction time, as it provides information needed for decision-making so that the next process can progress safely. SAs can monitor the strength development of concrete at the curing stage, and monitor the existence and severity of damage during the service life of concrete structures [112]. The typical experimental set-up of wave propagation (WP) technology is shown in Figure 9. The configuration consists of a laptop computer equipped with arbitrary waveform generation software, which allows users to generate transient waves and time spans of specific shapes and frequencies. An ultrasonic device was developed for damage detection and crack identification and was used to monitor the early age of concrete [113,114,115]. The main limitation of the device is that tests can only be carried out in a fixed design mold; therefore, on-site concrete monitoring is not permitted. Kim et al. [116] proposed a non-destructive curing strength gain monitoring method based on guided waves for early concrete, and derived a formula for estimating the curing strength of non-destructive tests [112].

Sun et al. [117] proposed a concrete strength monitoring method based on piezoelectric smart aggregates. The piezoelectric smart aggregate sensor was embedded in a concrete beam, and a signal monitoring test and a cube compressive strength test were carried out for each target age at 28 days. Through their experiments, Sun et al. drew four conclusions. Firstly, the energy of a piezoelectric smart aggregate monitoring signal attenuates with increasing concrete strength. Secondly, there is an approximately linear relationship between the energy ratio of the piezoelectric smart aggregate monitoring system and the percentage of concrete strength. Thirdly, when the parameters of the concrete specimen size and the monitoring signal are the same, the monitoring results can reflect differences in concrete strength grade. Finally, for the same concrete strength, the difference in specimen size and signal frequency will cause slight differences in monitoring results.

### 5.6. Monitoring Impact in Bridges

Impacts strongly affect the stability of bridge structures. However, without knowing the extent or direction of an impact on a pile foundation, it is difficult to estimate the damage caused and how to address it. Therefore, it is particularly important to have a precise way of monitoring the direction and extent of an impact.

Piezoelectric sensors were embedded in a road by Li et al. [118] to check the traffic flow and impact response of overloaded vehicles. Song et al. [119] used SA manufacturing of piezoelectric ceramic patches to monitor highway bridges impacted by overloaded vehicles. Seydel et al. [120] connected piezoelectric sensors to composite plates to monitor stress time-history curves to determine impact loads. Zhou et al. [121] used piezoelectric sensors to identify the in-plane position of an impact load on a composite plate. Choi et al. [122] proposed a method for identifying impact loads that used distributed built-in sensors to detect the influence of external objects. The estimated value of the response comparator from the system model and its recognition system can be used to predict the location and force history of impacts. Ciampa et al. [123] proposed an algorithm for locating impact sources, and used six surface acoustic emission piezoelectric sensors to determine the bending speed of Lamb mode A0. Kim et al. [124] used PVDF and PZT sensors to monitor the low-velocity impact damage of composites.

**Figure 10:**
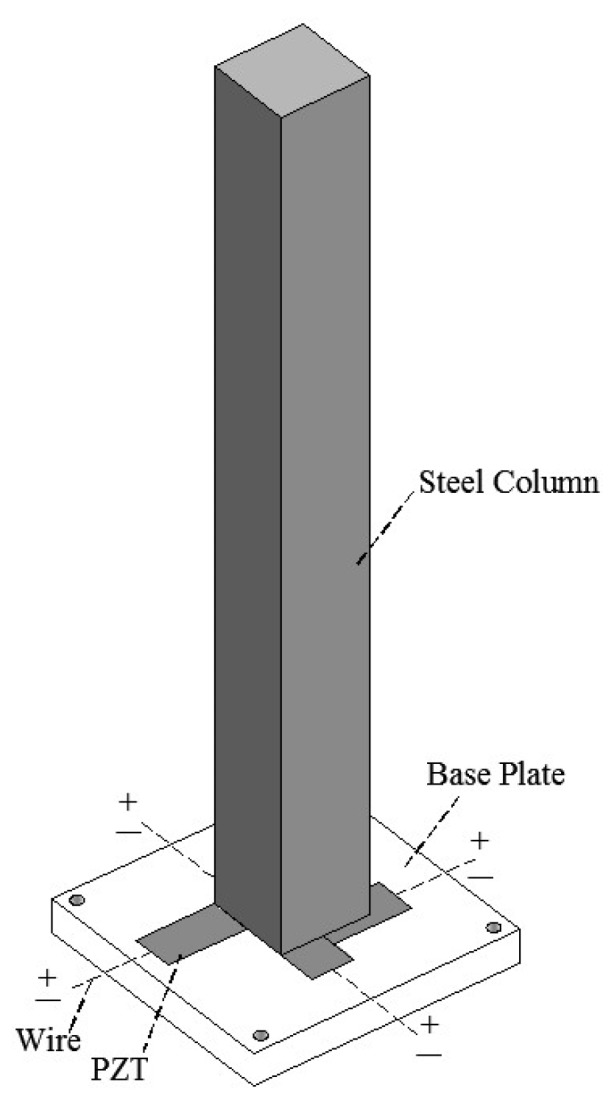
Diagram and photo of the impact direction sensor and PZT array [125].

Huo et al. [125] proposed a method for determining the direction of impacts based on the beat signal. The effectiveness of the method was verified by impact tests performed on a steel column with PZT transducers, as shown in Figure 10. The results showed that the PZT transducer could obtain beat signals caused by free vibration along two orthographic directions with similar frequencies. Based on the envelope of the beat signals, the direction of an impact could be identified effectively.

### 5.7. Monitoring Grouting Density in Bridges

After grouting, a reinforcement pipe can effectively prevent the corrosion of steel bars, maintain the bond performance between steel bars and concrete, and improve the bearing capacity of concrete structures. However, in practical applications, the grouting of post-tensioning tendons is consistently a problem of quality, such as the incomplete reinforcement of a tube, which leads to hollow or inefficient protection of steel tendons. Such deficiencies may reduce the integrity and service life of structures, possibly resulting in accidents. In the long run, water may enter the tendon canal in the gap, resulting in the corrosion of the tendons [71,126,127]. Therefore, to ensure the durability of reinforced concrete structures with post-tensioning tendons, the concrete must be fully filled in the passage to prevent water invasion and steel tendon erosion. However, monitoring grouting density remains a challenge because the grouting pipe is not visible in the grouting process [128].

**Figure 11:**
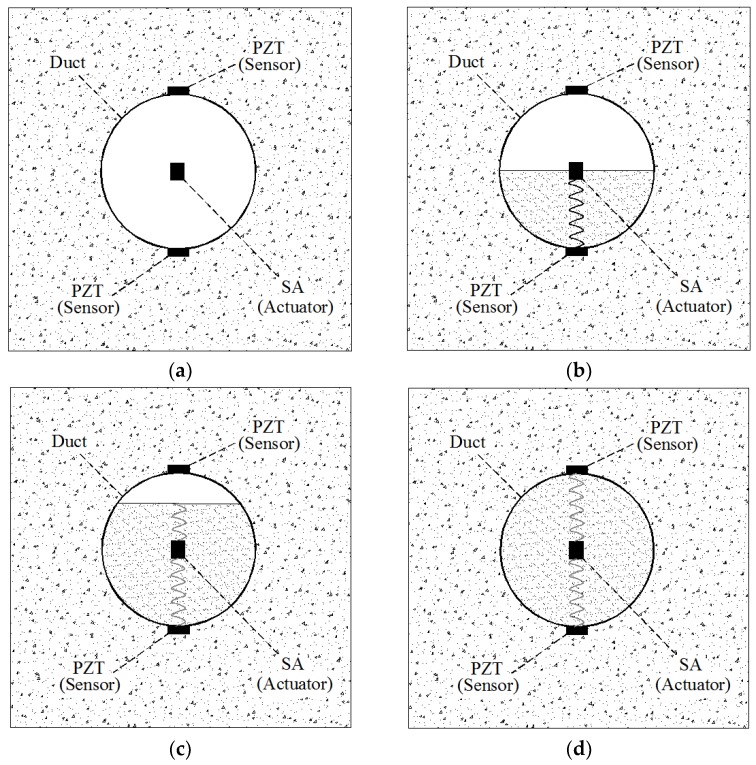
Principle of active sensing approach in monitoring grouting compactness: (**a**) empty duct; (**b**) half grouting; (**c**) 90% grouting; (**d**) full grouting [128].

Jiang et al. also proposed a method for monitoring the degree of grouting compaction in real time based on a stress wave piezoelectric ceramic sensor. In their study, a segment of a tendon was used as the object of research. The experimental process simulated four stages of grouting, including the empty state, half grouting, 90% grouting, and complete grouting. The experimental results showed that, when the PZT sensor formed a pipe between the SA and the PZT sensors, the PZT sensor at the bottom could detect a signal when the grouting level increased to 50%. The PZT sensor at the top could not receive any signal until the grouting process was completed (As shown in Figure 11). In their study, the authors used an energy analysis method based on wavelet packets to calculate the total signal energy received by PZT sensors. The experimental results showed that the energy level of the PZT sensor could reflect the grouting density in a pipeline. The proposed method may be used to monitor the grouting density of pre-stressed anchorage cables reinforced using the post-tension method [128].

To further understand the propagation of stress waves caused by piezoceramics in post-tensioning tendon ducts (PTTDs) with different grouting levels, Jiang [129] proposed a two-dimensional finite element model using a piezoceramic transducer to monitor the tightness of a tendon. Experimental verification showed that the proposed two-dimensional finite element model has the potential to be used to simulate the stress wave propagation principle to monitor the grouting and compaction of post-tensioning pre-stressed pipes.

### 5.8. Monitoring of Welding Problems in Bridges

Resistance spot welding (RSW) is widely used in modern industries and is one of the most effective techniques applied in plate connection. RSW is widely used in bridges, such as at connection nodes [130,131,132]. If spot welding failure occurs, the carrying capacity of the adjacent structure will be directly reduced; thus, such failure must be monitored in real time. However, due to the complexity and invisibility of spot-welded joints, the real-time health monitoring of such joints remains a challenge.

Ping Yao et al. [133] proposed a PZT active sensing method for the real-time monitoring of the structural health of multi-point-welded joints. Xu et al. [134] developed a piezoelectric transducer based on the high-frequency SH1 mode, which can be used to detect defects in long welds. Kim [135] proposed a method for monitoring the health of welded components in marine and offshore structures by measuring impedance data from piezoelectric ceramic materials to improve structural safety. Giurgiutiu et al. [136] detected defects in welded joints using piezoelectric ceramic transducers. Annamdas et al. [137] used EMI technology to study the load and non-load weld of fatigue-loaded welded beams. Rahman et al. [138] analyzed the effect of spot welding and thickness on the fatigue life of spot-welded joints via finite element analysis.

### 5.9. Monitoring Prestress-Loss

For bridges, the pre-stressing system (tendon anchorage) is a critical component that should be monitored regularly to ensure the performance of the bridges. In recent years, the practical application of piezoelectric transducers to detect pre-stressed concrete structures also increased.

Nguyen et al. [139] conducted wireless impedance-based pre-stress loss monitoring in tendon- anchorage connections using a PZT interface. In order to analyze the influence of pre-stress, a tendon-anchorage analysis model of the relationship between pre-stressing and structural parameters of the anchoring contact zone was established. Huynh et al. [28] used a portable PZT interface to monitor the impedance characteristics from the cable-anchorage subsystem for tension monitoring. Kim et al. [140] also proposed an impedance-based technique that used an installable PZT interface for pre-stress loss monitoring in local tendon anchors. A method to compensate for the influence of temperature change on impedance response was proposed, which makes the result of monitoring pre-stress loss more accurate. Huynh et al. [141] performed a numerical analysis of the local dynamics of the installable PZT interface to verify their feasibility of impedance monitoring for pre-stress loss in the tendon-anchoring subsystem. 

In addition to the use of PZT intelligent interfaces, the experimental study of pre-stressed monitoring of pre-stressed concrete beams based on piezoelectric impedance by Guo et al. [142] utilized externally attached PZT. Then, they used the statistical indicator RMSD to quantify the change in EMI spectra caused by the change of pre-stress, established the relationship between pre-stress and RMSD index, and monitored the pre-stress variation of the beam. Guo et al. [143] also conducted a monitoring test on the pre-stress of pre-stressed concrete beams when the pre-stressed values and pre-stress losses of the pre-stressed concrete beams were not sure. However, this was only a preliminary discussion, and further testing is needed in the future for quantitative analysis.

Steel strands are widely used in suspension bridges and cable-stayed bridges. Maintaining the safety and stability of steel strands is an important issue during bridge operation. Steel strands are subject to various types of pre-stress loss, and the loose wedge anchoring system can have a negative impact on the stability of the structure and even lead to accidents. Zhang et al. proposed a time reversal method that uses PZT patches as sensors and actuators to monitor the loose state of the wedge anchor system through active sensing based on stress waves [144].

## 6. Discussion

In the monitoring of bridges, piezoelectric ceramic materials have a wide range of applications; still, some problems remain to be solved. First of all, current piezoelectric ceramics used in the performance monitoring of bridge structure performance are basically completed in a simple and stable laboratory. Some scholars considered the influence of some environmental factors and made some compensations for the monitoring results to make it more accurate, such as temperature compensation for electromechanical impedance. However, the actual engineering environment of the bridge structure is a lot more complicated than that in the laboratory. Temperature changes, noise effects, wind and snow dust, and sensor installation methods all affect the monitoring effect. Therefore, how to consider the interference factor in the actual engineering environment is one of the main problems faced by the future piezoelectric ceramics for the monitoring of bridge structures.

Since bridge monitoring is a continuous long-term process, the design life of bridge engineering is at least 100 years. However, the working life of piezoelectric ceramic sensors is still unknown [61]. As Huynh et al. [145] stated in the article, because monitoring is a continuous and long-term process, even lasting decades for some bridges, the effectiveness of piezoelectric materials must be monitored throughout their life cycle. Therefore, further research is needed regarding the service life of piezoelectric ceramic sensors.

Moreover, the current research on piezoelectric ceramic sensors for bridge monitoring is mostly based on small-scale components or structural-scale models for research or experiment, and the size is much smaller than that of actual engineering components such as the studies in References [28,79]. Thus, the effect of piezoelectric ceramic sensors in practical applications requires further research. Additionally, piezoelectric ceramic sensors are used in bridge monitoring and are usually benchmarked. That is, the signal of the initial state is firstly measured as a healthy state, and the subsequently received signal is compared with the initial signal to determine the health state of the structure. In the future, it is necessary to further study the monitoring method without reference, and it is more convenient and quick to directly determine the stress state of the structure through the data of the sensor.

Next, in the piezoelectric ceramic monitoring based on the impedance method, very satisfactory results were obtained, for example, in References [65,66,67]. However, the impedance meter is very expensive and cannot be broadly applied in bridge structures. Therefore, it is very necessary to develop a small and inexpensive impedance analysis sensor, which can promote further development of bridge monitoring research.

In addition, the bridge structure has many parts which need to be monitored due to the complicated force. Field wiring is very complicated when using piezoelectric ceramic sensors for monitoring. Therefore, it is necessary to promote a piezoelectric ceramic wireless sensing system.

## 7. Conclusions

With increasing attention dedicated to bridge monitoring, the application of piezoelectric materials is becoming increasingly extensive. Piezoelectric materials are divided into compression and shear materials, which are used to monitor bridge defects under different mechanisms. In addition, there are two methods of employing piezoelectric materials for bridge monitoring: external attachment and internal embedment. Externally attached sensors have a smaller detection range and are more susceptible to environmental interference than embedded sensors; however, the received signal is more intense. Embedded sensors can monitor the health of a structure throughout its life cycle and are widely used in bridge monitoring.

This paper comprehensively summarized the application of piezoelectric materials in bridge monitoring, including monitoring the strength of concrete, loose bolts, corrosion, grouting density, and other aspects related to the intelligent monitoring of bridge structures. For each problem, corresponding research on piezoelectric materials via different research methods was expounded. The data processing methods related to bridge monitoring were summarized, including the wave method, time inversion, acoustic emission, and the impedance analysis method, and related research on various methods was outlined. Finally, the current problems faced in bridge monitoring and future research directions were discussed.
